# Long-term reoperation rates following spinal fusion for neuromuscular scoliosis in nonambulatory patients with cerebral palsy

**DOI:** 10.1007/s43390-024-00878-z

**Published:** 2024-04-29

**Authors:** Christopher D. Seaver, Sara J. Morgan, Candice S. Legister, Casey L. Palmer, Eduardo C. Beauchamp, Tenner J. Guillaume, Walter H. Truong, Steven E. Koop, Joseph H. Perra, John E. Lonstein, Daniel J. Miller

**Affiliations:** 1 Research Department, Gillette Children’s, St. Paul, MN USA; 2 University of Minnesota Medical School, Minneapolis, MN USA; 3 Division of Rehabilitation Science, University of Minnesota, Minneapolis, MN USA; 4 Department of Rehabilitation Medicine, University of Washington, Seattle, WA USA; 5 Department of Orthopaedic Surgery, University of Minnesota, Minneapolis, MN USA; 6 Department of Orthopaedic Surgery, Gillette Children’s, 200 University Ave E, Internal Zip 490105, St. Paul, MN 55101 USA; 7 Twin Cities Spine Center, Minneapolis, MN USA

**Keywords:** Neuromuscular scoliosis, Spinal fusion, Reoperation, Nonambulatory, Cerebral palsy

## Abstract

**Purpose:**

To describe the incidence of reoperation and factors contributing to surgical revision within a minimum of 10 years after spinal fusion for scoliosis in patients with nonambulatory cerebral palsy (CP).

**Methods:**

We conducted a retrospective review of consecutive nonambulatory patients with CP who underwent primary spinal fusion at a single specialty care center with a minimum of 10 years from their index surgery (surgery dates 2001–2011). Causes of reoperation were classified as implant failure/pseudoarthrosis, surgical site infection (SSI), proximal junctional kyphosis, prominent/symptomatic implants, and implant removal. Reoperation rates with 95% confidence intervals were calculated for each time interval, and an actuarial survival curve was generated.

**Results:**

144 patients met inclusion criteria (mean age = 14.3 ± 2.6 years, 62.5% male); 85.4% had 5 years follow-up data; and 66.0% had 10 years follow-up data. Estimates from the actuarial analysis suggest that 14.9% (95% CI: 10.0–22.0) underwent reoperation by 5 years postsurgery, and 21.7% (95% CI: 15.4–30.1) underwent reoperation by 10 years postsurgery. The most common causes for reoperation were implant failure/pseudoarthrosis, SSI, and prominent/symptomatic implants.

**Conclusions:**

To our knowledge, this study is the largest long-term follow-up of nonambulatory patients with CP and neuromuscular scoliosis who underwent spinal fusion. Approximately 22% of these patients required reoperation 10 years after their index surgery, primarily due to implant failure/pseudoarthrosis, SSI, and prominent/symptomatic implants. Complications and reoperations continued throughout the 10 years period after index surgery, reinforcing the need for long-term follow-up as these patients transition into adulthood.

**Level of evidence:**

III.

## Introduction

Cerebral palsy (CP) is the most common neuromuscular disease in the pediatric population [1]. Nonambulatory patients with CP frequently develop scoliosis, likely related to neuropathic and/or myopathic defects that affect spinal alignment [1–3]. Untreated, the abnormal spinal curvature may progress, potentially resulting in adverse sequelae including pulmonary compromise, pelvic obliquity, pain, skin integrity concerns, functional impairment, and/or impaired seating tolerance [1].

The propensity of the curve to progress often drives the management of neuromuscular scoliosis. For growing children with curve magnitudes larger than 40–50°, surgical correction may be recommended to prevent the adverse sequelae associated with a progressive deformity [2]. Surgical treatment has been shown to improve quality of life for patients and their caregivers [2, 4]. Surgical goals vary based on ambulatory status, current level of function, and degree of curvature [2]. The current trend for deformity correction is through a posterior-only spinal fusion with segmental instrumentation [3, 5]. Simultaneous pelvic fixation is frequently utilized to address pelvic obliquity, which is often problematic in nonambulatory patients with neuromuscular scoliosis [3, 6, 7].

Spinal fusion can result in complications, which may include pulmonary and cardiovascular compromise, surgical site infections (SSI), neurological deficits, implant failure, hemodynamic instability, curve progression, prominent/symptomatic implants, and pseudoarthrosis [8–10]. Prior studies have reported 30-day complication rates following spinal fusion for neuromuscular scoliosis to be as high as 17.9%, which is higher than in patients with congenital (10.6%) and idiopathic scoliosis (6.3%) [11]. One potential ramification of postoperative complications is the need for surgical revision, which has been reported as being 4.4 times more likely for patients with CP compared to those with idiopathic scoliosis in the first 90 days after surgery [12]. Another short-term study of reoperation rates reported that 9.7% of patients with neuromuscular scoliosis underwent reoperation within a year of their index procedure [13].

While reoperation rates have been described within the first year following the index procedure, long-term rates of reoperation for patients with CP have not been reported. Our aim is to describe the incidence of reoperation and reasons for surgical revision 10 years after spinal fusion in nonambulatory children with CP and neuromuscular scoliosis. These findings will inform patient and caregiver counseling and facilitate shared decision-making when considering surgical intervention for these patients.

## Materials and methods

We conducted a retrospective cohort study to investigate the incidence and factors contributing to reoperation 10 or more years after spinal fusion surgery in children with nonambulatory CP and associated neuromuscular scoliosis at a single specialty center. This project was reviewed and approved by an Institutional Review Board [de-identified for review].

### Patients

Eligibility criteria were as follows: (1) diagnosis of nonambulatory CP resulting in neuromuscular scoliosis, (2) index spinal fusion surgery before 21 years of age, (3) index spinal fusion surgery between January 2001 and December 2011, (4) and documentation of clinical and radiographic data two years after index surgery. Index spinal fusion surgery dates were chosen to identify patients with the potential for 10-year follow-up. Patients with prior spinal deformity surgery, index surgery at a different site, and those who opted out of research activities were excluded.

To identify eligible patients, an initial list was created using diagnosis codes for CP and neuromuscular scoliosis and procedure codes for spinal fusion. Medical documentation for these patients was then screened to confirm eligibility.

### Procedure

Data for eligible patients were abstracted from the institution’s electronic medical record into a Research Electronic Data Capture (REDCap) database (Vanderbilt University, Nashville, TN) [14, 15]. Clinical data abstracted for this study included demographic data (e.g., date of birth, sex), preoperative clinical history (e.g., type of CP, Gross Motor Function Classification System (GMFCS) [16] and subclassification [17]), index surgery information (e.g., date of surgery, spinal instrumentation type, and postoperative outcomes (e.g., number of reoperations, reason(s) for reoperation). Types of spinal instrumentation among patients in the study included sublaminar wires, pedicle screws, hooks, or hybrid (a combination of sublaminar wires, hooks, and/or pedicle screws). Reasons for reoperation were classified as implant failure/pseudoarthrosis, SSI, proximal junctional kyphosis (PJK), prominent/symptomatic implants, and other. The most recent visit with either an orthopedic surgeon or physical medicine and rehabilitation (PM and R) physician was recorded to determine follow-up length and patients who were lost to follow-up. Both orthopedic and PM and R follow-up dates were included to account for the interdisciplinary team approach at our specialty center.

Radiographic outcomes were measured in a picture archiving and communication system (PACS) by a single, trained evaluator and documented in the REDCap database. All radiographs were seated films. Preoperative films were the most recent films taken before the index surgery. Postoperative films were taken 3–12 weeks after the index surgery at the first follow-up appointment.

### Analysis

Descriptive statistics were calculated for all variables across the total cohort and by study group [i.e., those with and without one or more reoperation(s)]. The distribution of all outcomes was assessed for normality using histograms and quantile–quantile plots. Key demographics and baseline clinical characteristics were compared between study groups using chi-square tests, Fisher exact tests, or Mann–Whitney–Wilcoxon tests as appropriate for the data type. Initial reoperation was assessed at the following time intervals: < 3 months, 3 months– < 1 year, 1– < 2 years, 2– < 5 years, and 5– < 10 years. For the primary outcome of the reoperation rate, a survival curve was generated using the actuarial (life tables) method. Reoperation rates and 95% confidence intervals by follow-up time interval were calculated. Primary reasons for reoperation were reported for total reoperations and for each time interval. SAS v.9.4 (SAS Institute Inc., Cary NC) was used for all analyses. The alpha level was set at < 0.05 for all analyses and adjusted for multiple comparisons using a family-wise Bonferroni correction.

## Results

### Patients

**Fig. 1:**
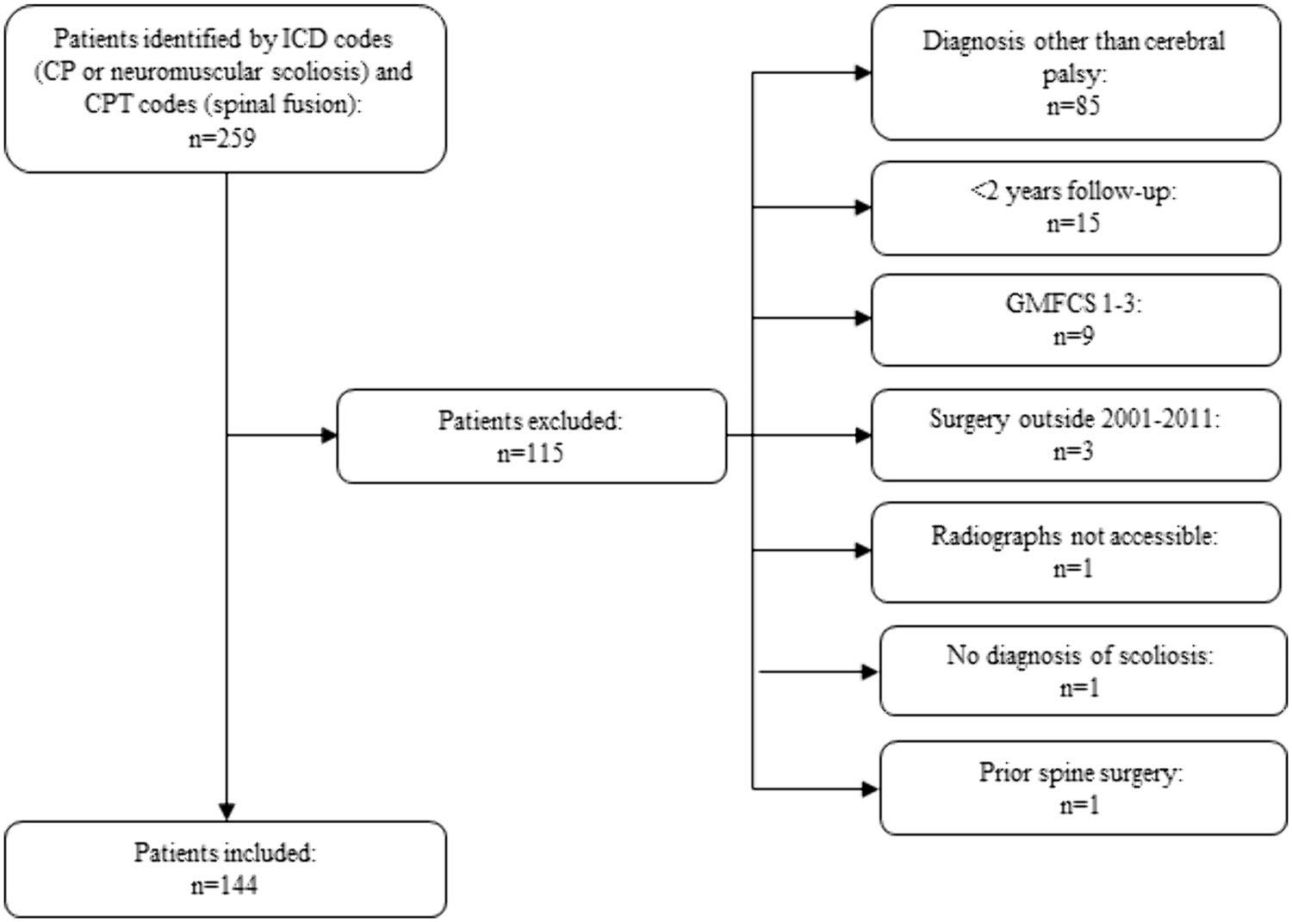
Flowchart of patients and reasons for exclusion from the study

A total of 259 patient records were identified (Fig. 1). Of those, 144 (55.6%) were eligible. Most ineligible patients had neuromuscular scoliosis, but their underlying diagnosis was not CP (e.g., myelomeningocele, muscular dystrophy). The average age was 14.3 ± 2.6 years, and 62.5% of the cohort was male (Table 1). Almost all patients had spastic-type CP (94.4%), and the pattern of involvement was almost exclusively quadriplegic (99.3%). Approximately 70% of patients had a preoperative GMFCS level 5; of GMFCS level 5 patients, most (54.4%) were subclassified as 5.3 [17]. Over half (55.6%) used a baclofen pump. The most common preoperative primary curve location was thoracolumbar (56.9%), and the average preoperative primary curve magnitude was 75.3 ± 22.7°. The average percent correction of the primary curve magnitude after the index fusion was 52.7 ± 20.2%. One-hundred and fifteen patients had an isolated posterior spinal fusion (79.9%), twenty-eight (19.4%) had a combined anterior and posterior approach, and one (0.7%) patient was treated with an anterior-only fusion. With respect to the type of spinal instrumentation, over half of the patients had sublaminar fixation (52.1%), followed by hybrid constructs (46.5%). Most patients (95.8%) also underwent pelvic fixation. (Table 2). Throughout the 10-year study period, a total of 10 (6.9%) patients died and 39 (27.1%) were lost to follow-up. Comparisons on demographic and clinical variables did not significantly differ between those that were and were not lost to follow-up (p-values ranged from 0.22 to 0.85). An additional three patients died in the 10 + year period (Table 3).

**Table 1:** Baseline demographic and clinical characteristics

	All participants *n* = 144			No reoperation* n = 115		Reoperation *n* = 29			*p*-value
	Mean	SD		Mean	SD	Mean	SD		
Age†	14.3	2.6		14.4	2.6	13.9	2.8		0.36
BMI†	16.8	4.8		16.6	5.1	17.7	3.6		0.14
	*n*	%		*n*	%	*n*	%		
Sex									0.49
Female	54	37.5		41	35.7	13	44.8		
Male	90	62.5		74	64.3	16	55.2		
Type of CP									0.66
Spastic	136	94.4		109	94.8	27	93.1		
Mixed	8	5.6		6	5.2	2	6.9		
Preoperative GMFCS									0.42
4	43	29.9		32	27.8	11	37.9		
5.0	5	3.5		3	2.6	2	6.9		
5.1	17	11.8		13	11.3	4	13.8		
5.2	24	16.7		21	18.3	3	10.3		
5.3	55	38.2		46	40.0	9	31.0		
Baclofen pump									0.53
Yes	80	55.6		62	53.9	18	62.1		
No	64	44.4		53	46.1	11	37.9		

*The “no reoperation” column includes participants without known reoperation

**Table 2:** Radiographic measurements and surgery characteristics

	All participants *n* = 144			No reoperation* n = 115		Reoperation *n* = 29			*p*-value**
	Mean	SD		Mean	SD	Mean	SD		
Pre-op primary curve magnitude	75.3	22.7		77.1	21.2	68.1	27.1		0.03
Post-op primary curve magnitude	36.1	18.3		37.0	18.2	32.5	18.5		0.31
Percent curve correction	52.7	20.2		53.2	19.4	51.0	23.6		0.64
Pre-op pelvic obliquity	20.2	37.0		20.5	40.9	19.2	12.7		0.54
Post-op pelvic obliquity	8.9	7.7		8.9	7.9	8.8	7.1		0.81
	*n*	%		*n*	%	*n*	%		
Primary curve apex									0.70
Thoracic	13	9.0		11	9.6	2	6.9		
Thoracolumbar	82	56.9		67	58.3	15	51.7		
Lumbar	49	34.0		37	32.2	12	41.4		
Spine fusion approach									1.00
Anterior	1	0.7		1	0.9	0	0.0		
Posterior	115	79.9		91	79.1	24	82.8		
Anterior and posterior	28	19.4		23	20.0	5	17.2		
Spinal instrumentation type									0.56
Pedicle screws	1	0.7		1	0.9	0	0.0		
Hooks (> 3 hooks)	0	0.0		0	0.0	0	0.0		
Sublaminar fixation	75	52.1		57	49.5	18	62.1		
Hybrid	67	46.5		56	48.7	11	37.9		
No instrumentation	1	0.7		1	0.9	0	0.0		
Pelvic fixation type‡									0.27
Sacral screws	2	1.4		2	1.7	0	0.0		
Iliac screws	31	21.5		25	21.7	6	20.7		
Iliac rods	90	62.5		73	63.5	17	58.6		
Hybrid	15	10.4		9	7.8	6	20.7		

*The “no reoperation” column includes participants without known reoperation

**α levels were adjusted using a Bonferroni correction to account for multiple comparisons; values < 0.005 were considered significant

^†^The values are given as the mean and the standard deviation

^‡^138 participants had pelvic fixation. 115 had no reoperation, and 29 had a reoperation

**Table 3:** Number of patients who underwent reoperation, died, or were otherwise lost to follow-up within each time interval

Time interval	Underwent reoperation	Died	Lost to follow-up
0– < 3 months	7	0	0
3 months– < 1 year	2	0	0
1 year– < 2 years	6	0	0
2 years– < 5 years	6	5	16
5 years–10 years	7	5	23
10 years or more	1	3	N/A

### Reoperations and estimated reoperation rates

**Fig. 2:**
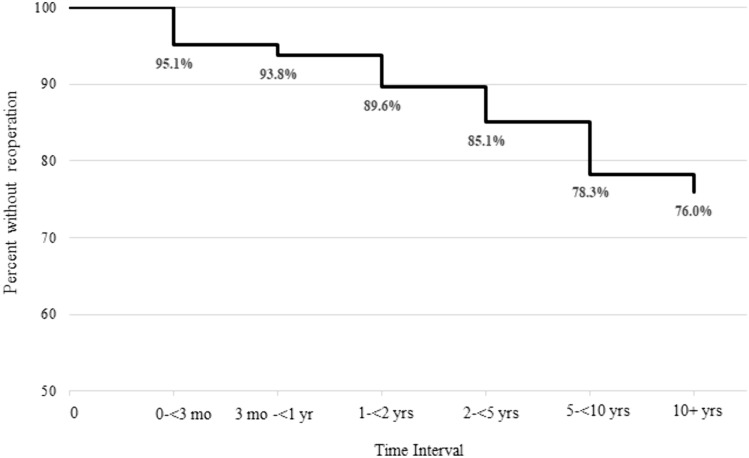
Survival curve based on the actuarial analysis of patients with cerebral palsy who underwent fusion surgery for neuromuscular scoliosis. This curve displays the probability that patients with neuromuscular scoliosis will not have a reoperation (i.e., survival) over a 10 years follow-up period following fusion surgery

Overall, 29 patients in this cohort had reoperations (Table 3). Seven patients had initial reoperations within the first three months of the index procedure, and two additional patients had initial reoperations between three months and one year. Roughly equal numbers of initial reoperations occurred in each of the subsequent time periods. Only one initial reoperation happened after 10 years. Estimated “survival” (i.e., not having a reoperation) rates at 5 and 10 years following the index surgery were 85.1% (95% CI: 78.0–90.0%) and 78.3% (95% CI: 69.9–84.6%), respectively (Table 4, Fig. 2).

**Table 4:** Survival (i.e., no reoperation) calculations from actuarial analysis

Time interval		Number at risk at start of the interval	Average number at risk during the interval	Survival** probability	95% CI
0– < 3 months		144	144	95.1%	90.1%, 97.7%
3 months– < 1 year		137	137	93.8%	88.3%, 96.7%
1 year– < 2 years		135	135	89.6%	83.3%, 93.6%
2 years– < 5 years		129	118.5	85.1%	78.0%, 90.0%
5 years–10 years		102	88	78.3%	69.9%, 84.6%
10 years or more		67	34	76.0%	66.4%, 83.2%

*There was 100% follow-up through the first two years by study design. At the end of the last time point, everyone is considered lost to follow-up

**Survival was defined as not having a reoperation by the end of the time interval

Of the 29 patients who had a reoperation, nine patients had *additional* reoperations. The most common reason for multiple reoperations was SSI; seven patients had between 2 and 14 total reoperations due to SSI. Two of the patients with multiple reoperations from SSI had recurrent SSI events that led to additional reoperations after the first year of follow-up. The remaining patients had additional reoperations due to prominent/symptomatic implants (*n* = 1) and implant failure/pseudoarthrosis (*n* = 1); these patients had a total of two reoperations each.

### Reasons for reoperation

Implant failure/pseudoarthrosis (34.5%) and SSI (34.5%) were the most common reasons for reoperation, followed by prominent/symptomatic implants (20.7%) (Table 5). All patients with implant failure were noted to have associated pseudarthroses on surgical exploration; patients with prominent/symptomatic implants and PJK had an associated concern that led to reoperation (e.g., pain, impaired seating, concerns with skin integrity, poor head positioning). Only one person had multiple reasons selected for reoperation (i.e., SSI and prominent/symptomatic implants).

**Table 5:** Reasons for initial reoperation across time intervals and by time interval

Reason for reoperation	Total			By time interval (*n*)					
	*n*†	%*		0– < 3mo†	3mo– < 1 yr	1– < 2 yr	2– < 5 yr	5– < 10 yr	10 + yr
Implant failure/pseudoarthrosis*	10	34.5		–	–	3	3	3	1
Surgical site infection	10	34.5		7	1	–	1	1	–
Prominent/symptomatic implants	6	20.7		1	1	3	0	1	–
Proximal junctional kyphosis (PJK)	2	6.9		–	–	–	1	1	–
Implant removal to facilitate surgery unrelated to spinal deformity	2	6.9		–	–	–	1	1	–

^†^Of the 29 patients who had reoperation, one patient had an initial reoperation in the 0- to 3-month time interval due to two reasons (SSI and prominent/symptomatic implants). Thus, 30 reasons are noted for initial reoperation across 29 patients

*Percentages are out of the number of patients (*n* = 29) who had reoperation and thus will add to > 100%

Reasons for reoperation varied by time period (Table 5). Most reoperations in the 0– < 3-month time period were due to SSI. Implant failure/pseudoarthrosis was the most common reason for reoperations occurring after one year. Reoperations due to prominent/symptomatic implants mainly occurred within two years of the index surgery. PJK and implant removal to facilitate other surgeries (unrelated to spinal deformity) were the least common reasons for reoperation, with two reoperations each between 2 and 10 years after the index surgery.

## Discussion

Our aim was to estimate reoperation rates and the factors contributing to surgical revision following spinal fusion in nonambulatory patients with CP. Study findings suggest that approximately 5% of patients have reoperation in the first three months after spinal fusion. In our cohort, most of these early reoperations were due to SSI. By 10 years, the estimated percentage of patients with reoperation increased to 21.7%, with implant failure/pseudoarthrosis and SSI as the leading causes for reoperation in our study. Of the patients in this cohort who underwent reoperation, most (69.0%) had only one reoperation in the 10-year follow-up period. The most common reason for *additional* reoperations was SSI.

To our knowledge, this study is the largest long-term follow-up of nonambulatory patients with CP and neuromuscular scoliosis who underwent spinal fusion. Long-term follow-up in this patient population can be difficult as some pediatric institutions are not able to follow children into adulthood or when they transition into different care environments. Our institution is well-suited to study long-term outcomes for patients with CP and neuromuscular scoliosis because we provide continuous care and follow-up throughout the lifespan of complex patients with childhood-onset conditions.

Our findings are similar to those from studies observing short-term reoperation rates in patients with neuromuscular scoliosis. In a two-year follow-up study on revision risk after pediatric spinal deformity surgery, Fruergaard et al. [18] reported an overall reoperation rate of 11.7% at two years for patients with neuromuscular scoliosis compared to our rate of 10.4%. In another study, Paul et al. [19] reported an overall reoperation rate of patients with cerebral palsy of 14.5% at four years compared to 14.6% at five years in our study. While relative distributions of causes for reoperation differ, we see similar trends across studies. Specifically, SSI is the leading cause of reoperation in short-term follow-up across studies, and implant-related reoperations (i.e., pseudarthrosis, implant failure) are more common in later follow-up periods. Fruergard et al. [18] and Paul et al. [19] are both administrative database studies that include data across multiple clinical sites, whereas our study included patient data from a single specialty center. These methodological differences resulted in a smaller sample for our study, but with additional contextual information about the study cohort from our medical records. Similar rates of reoperation across early time points in our study compared to larger, administrative database studies increase confidence in our long-term reoperation estimates.

There is a paucity of research for long-term outcomes in reoperations after spinal fusions in patients with nonambulatory CP, but longer-term follow-up is available for patients with idiopathic scoliosis who underwent spinal fusion, and these data can provide useful context for our findings. Historic studies before 2002 on long-term outcomes for patients with idiopathic scoliosis have reported reoperation rates of 9.2–19.0% with 5–8 years of follow-up [20–23]. More recent studies report lower reoperation rates (5.2–7.5%) after 5 years of follow-up [23, 24]. A recent 5-year multicenter reoperation survivorship study [24] reported lower reoperation rates for patients with idiopathic scoliosis compared to our study findings in patients with CP. At three months, 2.0% of patients with idiopathic scoliosis underwent reoperations compared to 4.9% of patients with CP in our study. Similarly, at five years, 6.1% of patients with idiopathic scoliosis underwent reoperation compared to 14.6% of patients with CP in our study. At three months, infection was the most common cause of reoperation in both studies, and after five years, implant failure/pseudarthrosis was the most common cause. While reoperation rates overall were higher in our patients with nonambulatory CP when compared to patients with idiopathic scoliosis, the causes of reoperation were similar between the two patient populations.

The GMFCS can be used to classify patients with CP by their mobility; levels 4 and 5 indicate patients who either mostly or always rely on wheelchairs for mobility [16]. A study by Jain et al. [17] proposed a subclassification (5.0, 5.1, 5.2, 5.3) for patients who were GMFCS level 5 by the presence of a gastrostomy tube, a tracheostomy, history of seizures, and verbal status. Patients with none of the impairments were classified as 5.0, and those with three or more impairments were classified as 5.3. Jain and colleagues found that patients with more impairments were more likely to experience major complications after spinal fusion [17]. However, in our study, patients with more impairments (i.e., higher GMFCS subclassifications) did not experience higher rates of reoperation.

Long-term follow-up studies are critical to understand how surgery affects children as they transition to adulthood. However, standard-of-care practice is evolving and thus findings from these long-term studies may not adequately reflect current practice. Patients in the current study had their initial surgery between 2001 and 2011. At that time, the most common fixation technique at our institution was the Luque–Galveston technique with all sublaminar wires (52.0%) or hybrid constructs (46.6%), which included a combination of sublaminar wires, hooks, and/or pedicle screws. Pedicle screw instrumentation is currently the most common instrumentation for posterior spinal fusion due to its ability to achieve stronger segmental fixation and the concept of three-column fixation [6, 25]. Thus, our findings may differ from long-term outcomes for present-day patients who receive all-pedicle screw instrumentation. There are limited data comparing reoperation rates between cohorts with cerebral palsy who received different spinal fixation techniques; however, two studies compared reoperation rates in cohorts of patients with idiopathic scoliosis. A retrospective study [26] of 50 patients, half with pedicle screw instrumentation and half with sublaminar wire instrumentation, reported no reoperations for either group at two years. Another study [27] compared reoperation rates between a cohort of patients (2008–2012) who primarily received all-pedicle screw fixation and prior cohorts with different fixation techniques. Investigators found that five-year reoperation rates did not significantly differ between cohorts; however, causes for the reoperation differed [27]. Together, these studies suggest that rates for reoperation for prior cohorts with sublaminar wires and hybrid constructs may still provide insight into rates of reoperation for current patients with pedicle screw fixation. Future research will assess reoperation rates and reasons for reoperation in contemporary cohorts with pedicle screw fixation.

The risk of reoperation is important when considering the overall benefit to patients who undergo spinal fusion and their families. Each surgery involves significant costs, time away from school and work, and risks of complications and further operations. To balance the required resources and risks inherent in spine surgery, prior research suggests that health-related quality-of-life indicators significantly improve for all parameters, indicating that patient lives improve following these procedures. Importantly, patients who reported the lowest values for health-related quality-of-life indicators demonstrated the largest improvements following spinal fusion [28]. Together, information about reoperation risk and health-related quality-of-life improvement potential will help physicians prepare patients and families for likely long-term outcomes after surgery.

### Limitations

This study included data collected retrospectively from clinical documentation, which may have been missing data. In addition, patients with less than 10 years of follow-up were not contacted to confirm if they had a reoperation. This study was conducted at a single specialty center, which may not be representative of patients with CP living outside of our area. Fixation techniques used during the study timeframe were primarily sublaminar fixation, which limits our ability to comment on the survivorship of all-pedicle screw constructs. Further research is needed to assess the generalizability of outcomes from current-day practices, and study findings can also be used as a baseline for future studies investigating long-term outcomes. Lastly, in this study, we defined success as not having a reoperation. The decision to undergo reoperation is complex; some patients with similar indications for a reoperation may elect to move forward, while other patients choose not to have the reoperation.

## Conclusion

Approximately 20% of patients who had spinal fusion for scoliosis associated with nonambulatory CP underwent reoperation 10 years after their index surgery. The reasons for reoperation were primarily due to implant failure/pseudoarthrosis, SSI, and prominent/symptomatic implants. Complications and reoperations continued throughout the 10-year period after index surgery, reinforcing the need for long-term follow-up as these patients transition into adulthood. This knowledge can be used to guide surgeons when counseling patients and families to set clear expectations and consent for surgery.
